# The temporal modulation structure of illiterate versus literate adult speech

**DOI:** 10.1371/journal.pone.0205224

**Published:** 2018-10-24

**Authors:** João Araújo, Sheila Flanagan, Alexandre Castro-Caldas, Usha Goswami

**Affiliations:** 1 Centro de Investigação Interdisciplinar em Saúde (CIIS), Universidade Católica Portuguesa, Palma de Cima, Lisboa, Portugal; 2 Centre for Neuroscience in Education, Department of Psychology, University of Cambridge, Cambridge, United Kingdom; Purdue University, UNITED STATES

## Abstract

The temporal modulation structure of speech plays a key role in neural encoding of the speech signal. Amplitude modulations (AMs, quasi-rhythmic changes in signal energy or intensity) in speech are encoded by neuronal oscillations (rhythmic variations in neural excitability in large cell networks) that oscillate at matching temporal rates. To date, however, all neural studies have investigated adult-directed speech (ADS) as produced and perceived by highly literate adults. Whether temporal features of ADS vary with the skills of the speaker, for example literacy skills, is currently unknown. Here we analyse the temporal structure of ADS spoken by illiterate, low literate (≤ 4 years of literacy) and highly literate (≥ 12 years of literacy) adults. We find that illiterates produce speech differently. Spontaneous conversational speech produced by illiterate adults showed significantly less synchronised coupling between AM bands (less phase synchronisation) than conversational speech produced by low literate and highly literate adults, and contained significantly fewer syllables per second. There was also a significant relationship between years of literacy and the amount of theta-band energy in conversational speech. When asked to produce rhythmic proverbs learned in childhood, all groups could produce speech with similar AM phase synchronisation, suggesting that the differences in spontaneous conversational speech were not caused by physiological constraints. The data suggest that the temporal modulation structure of spoken language changes with the acquisition of cultural skills like literacy that are usually a product of schooling. There is a cultural effect on the temporal modulation structure of spoken language.

## Introduction

Recent advances in our understanding of the neural basis of speech encoding suggest that neuronal oscillations at multiple timescales play a key role in encoding amplitude modulation patterns in speech, ‘multi-time resolution processing’ [1,2]. Adult MEG (magnetoencephalography) and EEG (electroencephalography) research reveals that the speech stream is sampled continuously by networks of cells that vary in endogenous excitability at different temporal rates. These cell networks use perceptual acoustic ‘landmarks’ in the speech signal such as amplitude rise times to phase-reset their oscillating activity to align with similar energy variations (such as amplitude modulations, AMs) in speech, thereby encoding the signal ([3], for review). Cell networks in auditory cortex form an *oscillatory hierarchy* [4], and speech intelligibility studies with ADS reveal a key role for neuronal oscillations in four temporal rate bands in this hierarchy, *delta*, ~1–3 Hz, *theta*, ~4–8 Hz, *beta*, ~15–30 Hz, and *low gamma*, ~30–50 Hz [5]. There is maximal modulation energy in ADS in the theta band, 4–8 Hz [2], and accordingly it has been proposed that theta entrainment is a core feature of speech encoding by adults [3].

All neural studies of the role of the temporal modulation structure of speech to date have utilised highly literate adults, typically university students. However, many users of spoken language in the real world are illiterate, usually because of lack of access to education. Accordingly, it is *in principle* possible that the features of ADS thought to govern neural processing, such as maximal theta band energy, are a product of literacy acquisition. Literacy acquisition is already known to change speech perception and phonological processing (processing of the sound structure of speech). While illiterate adults can perform as well as literate adults in phonological tasks such as rhyme identification, they are very poor at phonological tasks requiring the identification and/or manipulation of phonemes, such as phoneme deletion [6,7]. As illiterate adults have never been taught to read, they have never developed a specialised letter processing system. In child development, it is literacy acquisition that drives both the development of a specialised letter processing system and phoneme awareness [8]. Illiterate adults are also poorer at repeating nonsense words compared to non-illiterate adults [9], and at performing syllable awareness tasks. Illiterate adults are significantly slower in picture naming tasks than literate adults (293 ms on average, [10]), and show a different time-course for phonological priming of picture naming (e.g., auditory prime ‘Sede’, for a picture of the sun [sol]) [10]. Although the illiterate adults in [10] showed naming facilitation from the shared onset phoneme in the auditory prime, this was only the case when the picture was already displayed. For literate adults, phonological priming effects were present when the prime was presented 150 ms before the picture was displayed [10]. Learning to read also changes the neural organisation of the brain’s language areas [11–13]. For example, brain activation during oral language tasks varies as a function of literacy (see [14] for a recent review). Training in literacy improves phonological awareness at all linguistic levels, particularly phoneme awareness, while also developing to a lesser extent syllable and rhyme awareness [6, 15]). Indeed, literacy has been compared to a “virus” that affects every area of speech processing [16]). Accordingly, it is possible that becoming literate also affects the temporal modulation structure of produced speech.

To investigate this question, we built on modelling work that has enabled the comparison of the modulation structure of infant-directed speech (IDS) and ADS. Modelling of rhythmic child-directed speech (English nursery rhymes) revealed an AM hierarchy that mirrors the neural oscillatory hierarchy [17]. The AM hierarchy encompassed three broad bands of energy, AMs in a delta band (0.9–2.5 Hz) centred on ~2 Hz, AMs in a theta band (2.5–12 Hz) centred on ~5 Hz, and AMs in a beta/low gamma band (12–40 Hz), centred on ~20 Hz. The degree of phase alignment (rhythmic synchronisation) between the AMs in the two slower bands (delta-theta phase alignment) was related to whether the listener perceived strong (stressed) or weak (unstressed) syllables [18]. Subsequent comparison of IDS and ADS using this Spectral-Amplitude Modulation Phase Hierarchy (S-AMPH) model revealed that the AM hierarchy has different characteristics in each speech register [19]. Firstly, IDS was characterised by significantly more energy in the delta band compared to ADS. Secondly, ADS had significantly more energy in the theta band compared to IDS. Thirdly, the phase alignment of AMs in these three bands differed with speech register. For IDS, the phase alignment of AMs in the delta band with AMs in the theta band was significantly greater than in ADS. In contrast, for ADS, theta band AMs showed significantly greater phase alignment with faster beta/low gamma band AMs than in IDS. This was interpreted to show a more regular spacing of stressed syllables in speech used with infants, and a more regular spacing of phonemes in syllables in speech used with adults. Hence important differences are found in the temporal modulation structure of the speech produced by adults depending on the listener (infants versus literate adults). The most likely explanation is that these temporal modifications facilitate the acquisition of spoken language by the infant brain [19].

Given the well-documented effects of literacy acquisition on spoken language processing, it is plausible to suggest that the acquisition of literacy may also result in temporal modification of the speech signal. There are very few studies of speech production within an oscillatory perspective [20], and none to our knowledge with illiterate adults. There are some studies of speech production by adults with *reduced* literacy (adults with developmental dyslexia, e.g. [21]), but these have typically focused on phonetic features. The exception is a study of rhythmic speech (English nursery rhymes) produced by dyslexic versus non-dyslexic adults ([22]). The adults with dyslexia in [22] showed atypical theta-beta/low gamma phase synchronisation compared to non-dyslexic highly literate controls when producing nursery rhymes to a metronome beat, indicative of differences in syllable timing. There are also a number of studies with children with developmental dyslexia conducted within a neural oscillatory perspective. These studies have reported atypical encoding of slower AMs in the speech signal, in the delta band (0–2 Hz) [23,24]. Meanwhile, typically-developing children showed a significant relationship between theta entrainment and learning to read [25]. It is thus possible that the key role identified for theta-rate information in adult speech processing may be related to literacy development.

To test this hypothesis, we recorded conversational speech as spoken by age-matched adults who were either illiterate because of lack of access to schooling in early life (illiterate group), had acquired basic school literacy (~4 years of training; low literate group) or were university graduates (high literate group). If literacy changes the temporal modulation structure of conversational speech, then the AM structure of ADS produced by the high literate group would be expected to show differences when compared to the ADS produced by the low literate and illiterate groups. As a comparison condition, we also recorded the production of rhythmic proverbs that our participants would have learned by heart in childhood. Here there is a familiar rhythmic template that underpins speech production, so groups should produce speech with a similar temporal modulation structure. We analysed the modulation spectrum of the speech produced by each group, predicting no group differences for deliberately rhythmic speech, but group differences for conversational speech. We expected greater modulation energy in the delta band for the illiterate participants, and greater modulation energy in the theta band for the highly literate participants. We also expected differences in the strength of phase alignment between AM bands. When ADS was compared to IDS [19], phase alignment between theta- and beta/low gamma band AMs was stronger in ADS, indicating a more regular spacing of phonemes in syllables. Accordingly, we predicted stronger phase alignment between theta- and beta/low gamma band AMs in the conversational speech of high literates, as they will have acquired phoneme awareness via learning to read. Finally, we predicted relationships between the temporal modulation structure of conversational speech and success in phonological tasks measuring syllable, rhyme and phoneme awareness. Across languages, explicit awareness of phonemes is a cognitive consequence of learning to read [8]. Hence any temporal features that may differ between the conversational speech of illiterate and highly literate participants should be related to individual differences in phoneme awareness.

## Materials and methods

### Participants

This research was approved by Universidade Católica's Ethics Committee and was conducted under strict adherence to the guidelines for the use of human subjects at Universidade Católica Portuguesa. Forty-six adults aged between 69 and 91 gave informed consent (in written form for literates and orally for illiterates) regarding participation: 15 illiterates (2 males, mean age 80.4 ± 4.4 years), 19 “low literates” with 4 years of literacy instruction or less (2 males, mean age 77.9 ± 5.7 years) and 12 “high literates” with more than 12 years of literacy instruction (1 male, mean age 79.7 ± 6.3 years). All participants reported normal or corrected to normal eyesight and no history of neurological disease. A one-way ANOVA confirmed the absence of age differences across groups. To qualify as illiterate, a participant may not have attended school, have had any literacy training as an adult, and be unable to read any items on a word list (monosyllabic and disyllabic) presented prior to testing. To qualify as literate, reading and writing had to be learned in childhood [26]. To rule out possible cognitive decline or dementia, a self-report questionnaire of activities of daily living (IADL-B [27] adapted to Portuguese [28]) and a cognitive screening test (MMSE [29] adapted to Portuguese [30]) were administered. Self-reported incapacity to perform more than one of the daily activities in the IADL-B and/or a score on the MMSE below the cut point (as in [30]) would exclude the participant from this study.

### Phonological awareness, vocabulary and general verbal ability tasks

All the testing sessions took place in quiet rooms (usually small offices or living rooms) with soft furnishings. The participants performed phonological awareness tests designed for this study:

1. Syllabic division task: segmentation of 1- to 3-syllable words under the guise of a tapping game, following the paradigm in [31] for non-literate populations (Maximum score: 24 points). The target words were very frequent European Portuguese words and contained V, CV, VC, CVC, CCV and CCVC syllables.
2. Rhyme detection task: deciding whether pairs of words matched for frequency, neighbourhood density and length rhymed (forced-choice task, maximum score: 24 points).
3. Phoneme deletion task: deletion of the first phoneme of mono- and di-syllable words whose frequency (based on the European Portuguese corpus LMCPC available at http://www.clul.ul.pt) and neighbourhood density (based on the Porlex database [32]) were manipulated orthogonally (Maximum score: 32 points).

Feedback was provided for the first two items of each task. As illiterates consistently fail phoneme awareness tasks [6], the phoneme deletion task ceased after 5 consecutive errors to avoid demotivation. Receptive vocabulary was assessed using the vocabulary subtest from the WAIS-III [33] (translated and adapted to European Portuguese in [34]). A measure of speech comprehension was obtained using the Token Test often used in studies of aphasia [35] (version of 22 items translated and adapted to European Portuguese in [36]). Group data for all tests are shown in Table 1.

**Table 1:** Participant characteristics^a^.

	Illiterates n = 15	Low Literates n = 19	High Literates n = 12
**Age (years)**	80.4 (4.4)	77.9 (5.7)	79.7 (6.3)
**Literacy (years)**	0	3.3 (0.75)	15.5 (2.47)
**Syllabic division**	11 (3.4)	19.7 (4.3)	23.8 (0.5)
**Rhyme detection**	12.8 (3.2)	16.1 (3.8)	21 (3.1)
**Phoneme deletion**	0	22.6 (6)	30.1 (2.4)
**Vocabulary (WAIS-III)**	9.7 (3.6)	26.5 (11.5)	54.8 (5.7)
**Token Test**	11.8 (3)	16.6 (2.7)	19.5 (1.7)

^a^ SD in brackets

### Semi-structured interview and repetition of rhythmic utterances

To acquire samples of conversational speech, a semi-structured interview was conducted and recorded for 10–15 minutes. For speech recordings, a cardioid microphone (Audio-Technica ATR2100-USB) was used. The microphone was connected via USB to a portable computer with the software Audacity (http://audacityteam.org/about/). Speech samples were digitally recorded at 44.1 kHz. Three interview topics were chosen from those known to engage older adults [37]. They were 1) career and life experiences, 2) weekly activities and 3) relationships with family/friends. The experimenter used the following open-ended question: “Tell me about your [topic]”. As in [38] the experimenter changed the topic when the participant made a topic closing statement such as “that’s it” or “that’s everything” followed by silence (> 3 s), stopped speaking (> 3 s) or indicated in another manner that the topic or his/her turn was complete.

To acquire rhythmic speech, a proverb repetition task was administered. Portuguese proverbs, much like English proverbs, have specific rhythmic properties created by the use of alliteration, assonance, consonance and rhyme (see [39]). Proverbs were carefully selected to match specific metrical syllabic patterns (like the nursery rhymes in [17]). Therefore, they could be trochees (strong-weak syllable alternation, for example “Muito riso pouco siso”–strong syllables underlined), iambs (weak-strong syllable alternation, for example “Quem não arrisca não petisca”) or dactyls (strong-weak-weak syllable alternation, for example “Vozes de burro não chegam ao céu”). A total of 6 proverbs were repeated by each participant. The proverbs chosen are well known in Portuguese and no participant showed any difficulties in repeating them.

### Speech data processing

Before being analysed, the recordings were manually divided into shorter segments (containing a complete phrase or a proverb) using Audacity software. Illiterates contributed 89 segments, low literates 107 segments and high literates 90 segments of conversational speech in a total of 286 speech segments. Segments ranged from ~4s to ~20s and each participant contributed, on average, 6.2 segments (range 3 segments– 11 segments), plus 6 proverbs ranging from 1s to 3s each. Each speech segment was z-scored to standardise its mean and standard deviation and the Spectral-Amplitude Modulation Phase Hierarchy (S-AMPH) model [16] was applied. In the S-AMPH, the z-scored acoustic signal is band-pass filtered into five frequency bands (channel edge frequencies: 100, 300, 700, 1750, 3900 and 7250 Hz) using a series of adjacent finite impulse response (FIR) filters. Next, the Hilbert envelope is extracted from each band-filtered signal and the five Hilbert envelopes are down-sampled to 1050 Hz and passed through a second series of band-pass filters in order to isolate the three different AM bands within the envelope modulation spectrum. These three AM bands corresponded to delta rate modulations (0.9–2.5 Hz), theta rate modulations (2.5–12 Hz) and beta/low gamma rate modulations respectively (12–40 Hz).

To assess the temporal structure of the speech signal, two measures were derived from the S-AMPH model: the envelope modulation spectrum and a multi-timescale synchronization index. To calculate the modulation spectrum in each speech sample, the sub-band Hilbert envelopes of the stimuli were individually passed through a modulation FIR filterbank with 24 channels logarithmically-spaced between 0.9–40 Hz. The mean power across all modulation channels was computed for each frequency sub-band and the relative power difference from this mean was computed for each modulation channel. The differenced modulation power spectrum was then averaged across the 5 frequency sub-bands for each speech sample of every participant across the three literacy groups.

To measure signal energy across literacy groups, the area under the curve corresponding to the three modulation bands across the 5 spectral bands was computed. A measure of multi-timescale phase-synchronisation, the Phase Synchronisation Index (PSI) was computed between the nested modulation bands in the S-AMPH representation of each speech segment (i.e., delta-theta phase synchronisation and theta-beta/low gamma phase synchronisation). The PSI was computed as:

$$PSI=|\left\langle e^{i(n\vartheta_{1}‑\vartheta_{2})}\right\rangle |$$ (1)

In (1), *n* is an integer related to the frequency relationship between the two AMs being compared. Following [17], for the delta-theta AM band analysis, an *n* of 2 was used, while for the theta-beta/low gamma AM band analysis, an *n* of 3 was used. The values θ1 and θ2 refer to the instantaneous phase of the two AMs at each point in time. Therefore, (*n*θ1 - θ2) is the generalised phase difference between the two AMs, which was computed by taking the circular distance (modulus 2π) between the two instantaneous phase angles. The angled brackets denote averaging of this phase difference over all time-points. The PSI is the absolute value of this average, and can vary between 0 (no synchronization, typical of a sound random in rhythm) and 1 (perfect synchronization, typical of a sound with perfect rhythmic regularity). Delta-theta and theta-beta/low-gamma PSIs across the 5 spectral bands were averaged in every speech segment and a grand mean of the PSI values across speech segments was calculated for each participant.

In addition, measures of speech rate were computed for each participant. Speech rate was calculated as follows: A native Portuguese speaker transcribed each utterance and counted how many syllables were produced in every sound file. The rate of speech in a specific sound file was calculated by dividing its number of syllables by its total duration in seconds. The speech rate of each participant was then obtained by averaging the speech rate (syllables/second) of all his/her utterances. Conversational and rhythmic speech rates were calculated separately for each participant.

### Correlational analyses

Potential relationships between literacy, phonology, vocabulary and the modulation characteristics of conversational speech were analysed using Spearman correlations by rank.

## Results

Conversational speech rates differed across groups [F(4,84) = 6.877; p < .0001; Wilk’s Λ = .57; partial η2 = .246 ]. Tukey tests showed that high literates produced more syllables per second than illiterates (p = .001) and low literates (p < .001). This would be expected, as a group of highly literate individuals are likely to produce more complex syntax and polysyllabic words [40, 41]. No differences in speech rate were found when comparing low literates and illiterates (p = .52). No significant group differences were found for rhythmic speech rates across groups (all p’s >.14).

**Fig 1:**
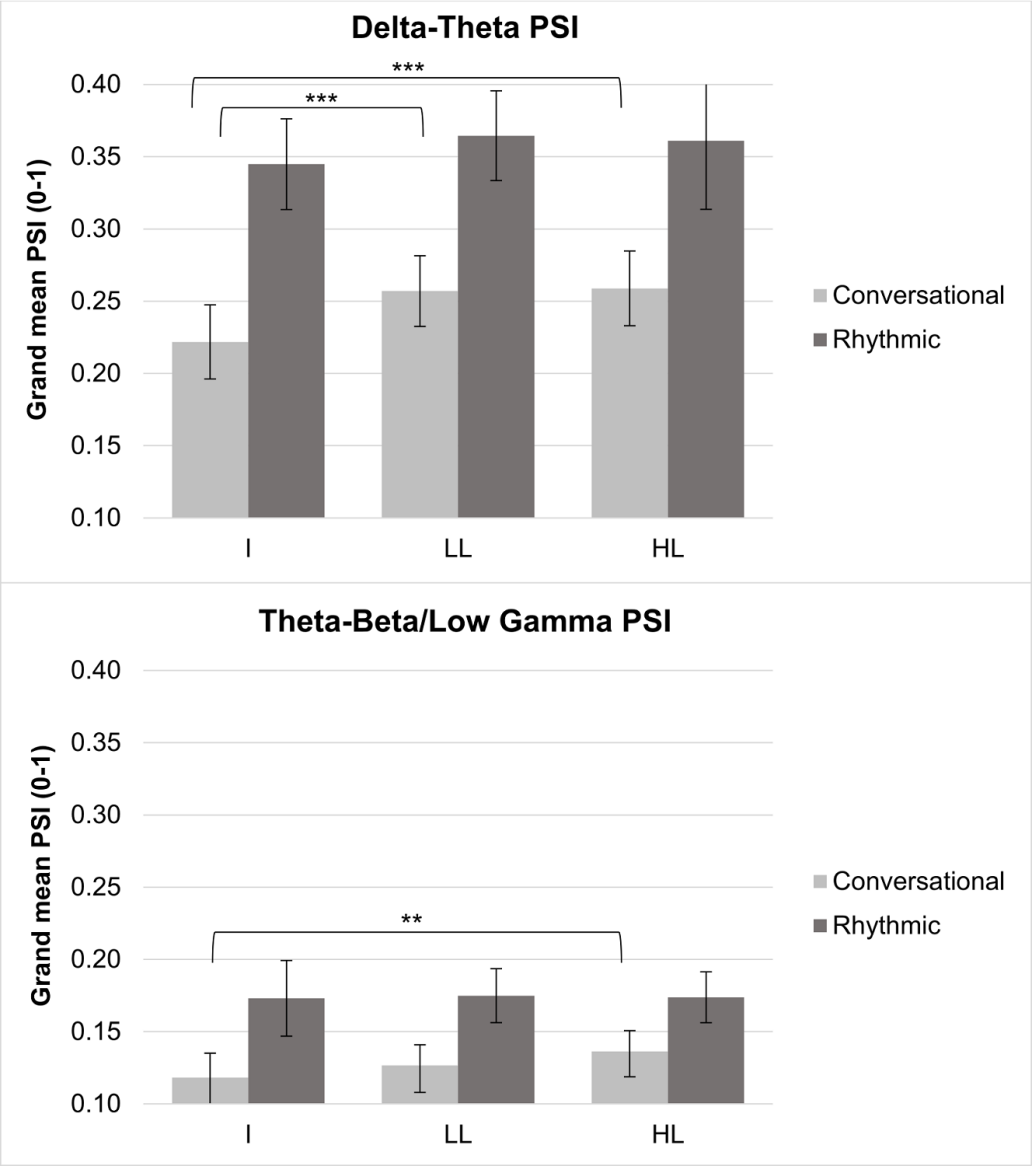
Grand mean PSI values for the two AM band pairs for rhythmic and conversational speech across the 3 literacy groups. I = Illiterates; LL = Low Literates; HL = High Literates. *p< .05; ** p < .01; ***p < .001.

**Fig 2:**
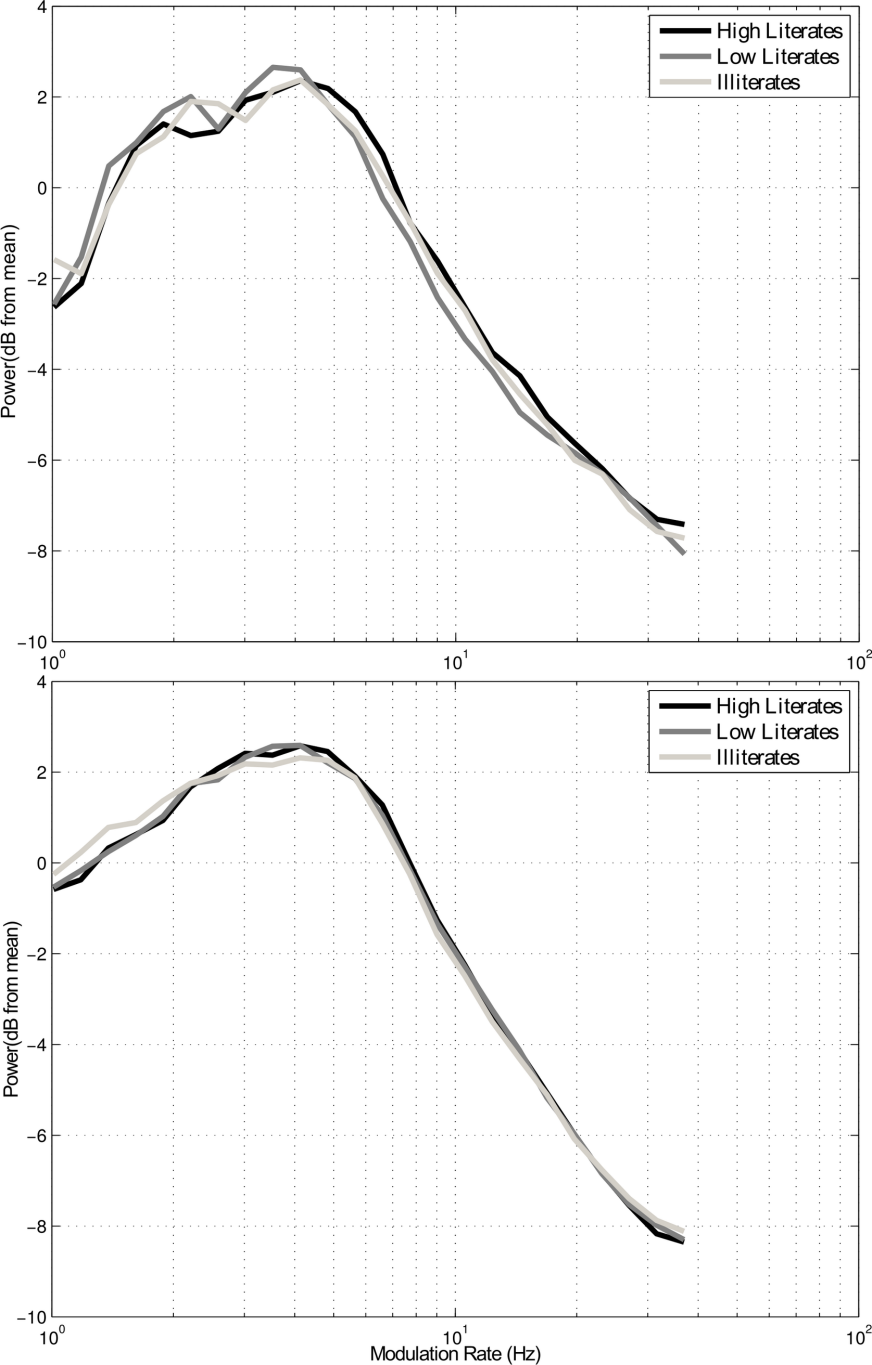
Grand mean modulation spectra calculated across the five S-AMPH spectral frequency bands for rhythmic and conversational speech across the different literacy groups. I = Illiterates; LL = Low Literates; HL = High Literates. Top panel = rhythmic speech, bottom panel = conversational speech.

Using the modulation phase synchronisation analysis approach in [17], we found group differences in the strength of both delta-theta AM phase alignment and theta-beta/low gamma AM phase alignment for conversational speech, shown in Fig 1. Contrary to prediction, the modulation spectrum analyses did not reveal significantly greater modulation energy in the delta band in the conversational speech of illiterates (Fig 2, bottom panel). A repeated-measures ANOVA taking the energy in each AM band as the dependent variable revealed no significant effect of group [F(2,43) = .734, p = .486 ] nor group x band interaction [F(2,43) = 1.547, p = .224 ].

To explore the effects of literacy on phase synchronisation, and given the speech rate differences for conversational speech but not rhythmic speech, separate MANOVAs were computed for conversational speech and rhythmic speech respectively. In each case, delta-theta PSIs and theta-beta/low gamma PSIs were the dependent variables, with literacy group as the independent variable. The MANOVAs revealed an effect of literacy in conversational speech for both the magnitude of delta-theta and of theta-beta/low gamma phase synchronisation [F(4,84) = 5.890; p = .00032; Wilk’s Λ = .61; partial η^2^ = .219]. Post-hoc Tukey tests showed lower delta-theta AM phase synchronisation for illiterates when compared to both low literates (p < .001) and high literates (p = .001), and also lower theta-beta/low-gamma phase AM synchronisation between illiterates and high literates (p = .01), but not between illiterates and low literates (p > .2). This suggests that literacy affects the regularity of the temporal spacing of different phonological units in conversational speech. No group differences were found for the MANOVA for rhythmic speech [F(4,84) = .697; p = .60 ], showing that all participating adults could produce temporally synchronised speech if required. Accordingly, there were no physiological constraints underpinning the differences in temporal modulation structure found for conversational speech.

**Fig 3:**
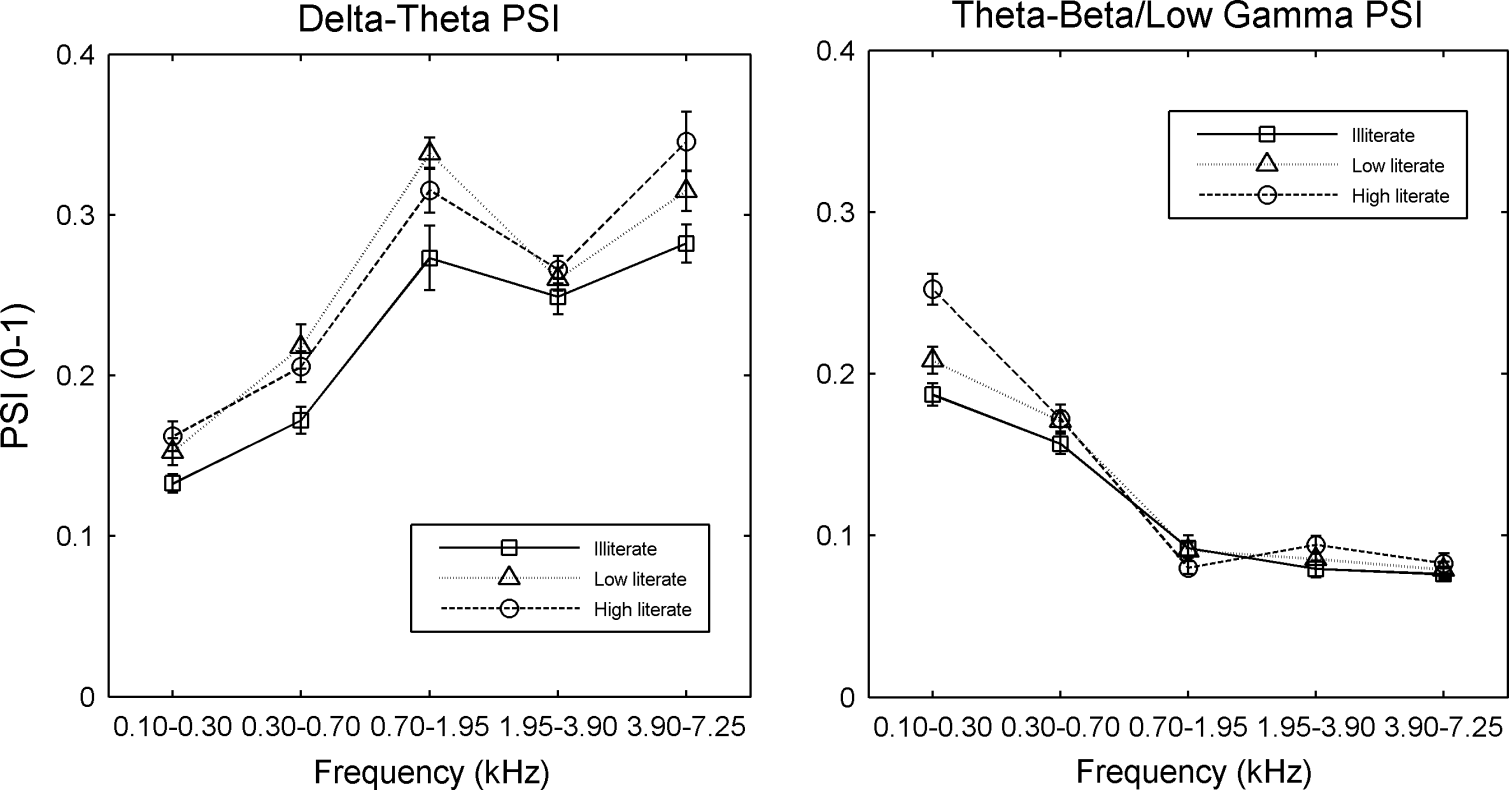
**Phase synchronisation index (PSI) values by frequency band for delta-theta amplitude modulation bands (left panel) and theta-beta/low gamma amplitude modulation bands (right panel) for conversational speech recorded from illiterate, low literate and high literate adults.** The *x-axis* indicates the spectral bands from which the amplitude modulations originate. The *y-axis* shows the PSI value. Error bars are in standard error of the mean.

An additional analysis was carried out for both the conversational speech data and the rhythmic speech data, to see whether the group differences in phase synchronisation shown in Fig 1 were consistent across the different frequency bands in speech. Two repeated-measure 3 x 2 x 5 ANOVAs were run, one for conversational speech and one for rhythmic speech, with AM Band Pair (delta-theta, theta-beta/low gamma) and spectral Frequency Band (bands 1–5) as repeated measures, literacy as the between-group factor, and PSI values as the dependent variable in each case. Only the ANOVA for conversational speech showed a three-way interaction between Literacy, AM Band Pair and Frequency Band, F(8,172) = 2.24, p< .05, *ηρ^2^* = .094, suggesting differential effects by Frequency Band. The interaction was explored by running two individual ANOVAs, one for each AM Band Pair. The ANOVA for the slower band pairing (delta-theta PSI) showed a main effect of Literacy, F(2,43) = 10.1, p< 001, but no interaction between Literacy and Frequency Band. Post-hoc tests (Tukey) showed that the illiterate PSI was always of a lower magnitude than the low literate PSI (p< .001) and the high literate PSI (p< .001), while the PSI for high and low literates did not differ. This effect is shown in Fig 3. The ANOVA for the faster band pairing (theta-beta/low gamma PSI) did show a significant interaction between Literacy and Frequency Band, F(8,172) = 5.4, p< .001, *ηρ²* = .202. Post-hoc inspection of the interaction showed that it was carried by group differences in Band 1 only (this is the lowest frequency band, 100–300 Hz, corresponding to F0). Hence illiterate participants showed significantly less phase synchronisation than high literate (p< .001, post-hoc Tukey test) participants between faster AM bands in Frequency Band 1, also depicted in Fig 3.

**Fig 4:**
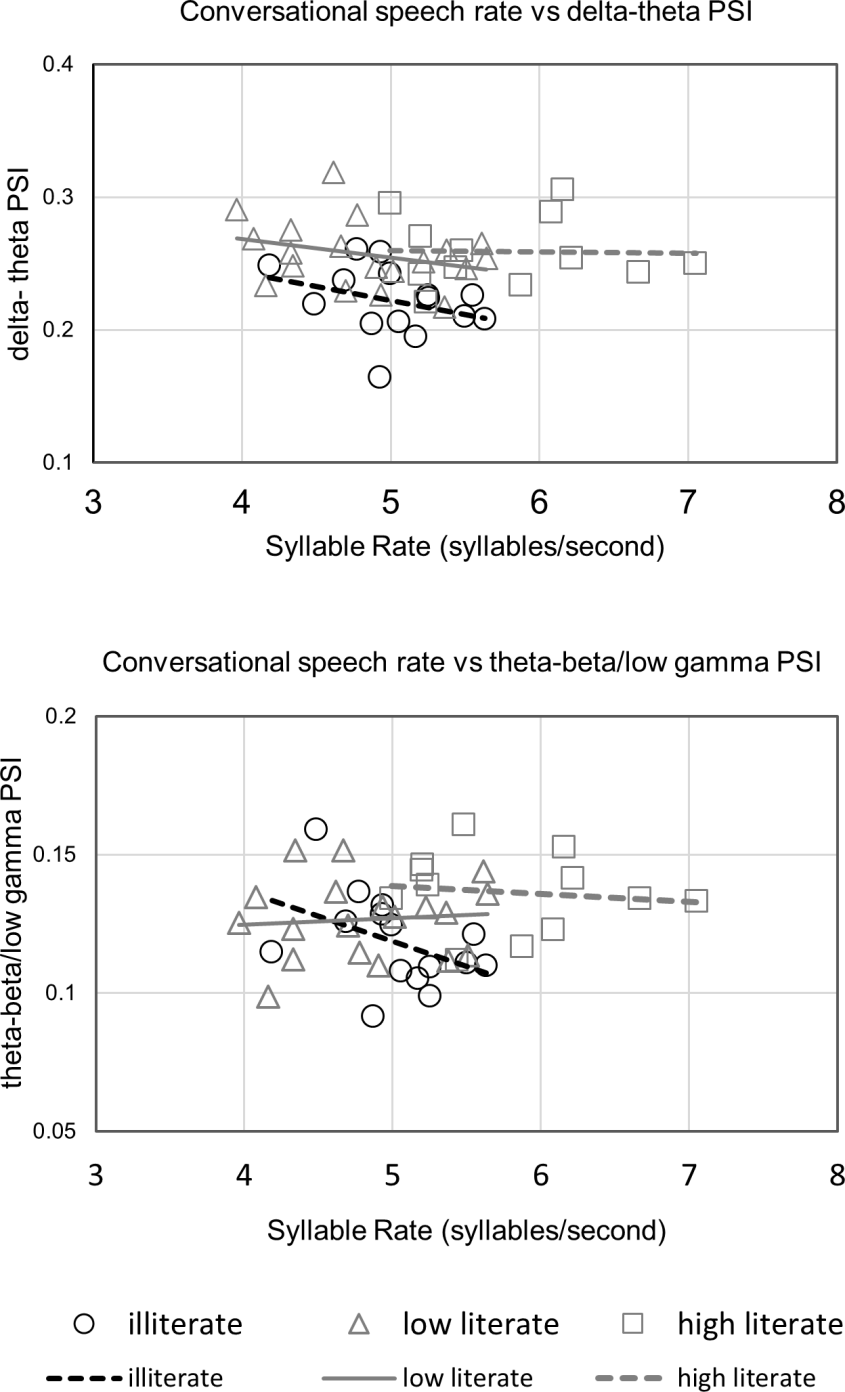
**Participant mean conversational syllable rate (syllables per second) plotted against Phase Synchronization Index(PSI) values for delta-theta PSI (upper panel) and theta-beta/low gamma PSI (lower panel), with linear trend lines depicted.** All correlations, p>0.05.

Finally, we investigated whether the faster average speech rate of the group of highly literate participants would affect the phase synchronisation analyses. We correlated conversational speech rate with mean PSI for the delta-theta PSI and the theta/beta-low gamma PSI mean values for each participant. The data were normally distributed (Shapiro- Wilk test for normality, all p’s> .05) and are shown in Fig 4. Pearson correlations showed no significant relationship between speech rate and PSI for any of the groups. The delta-theta PSI correlations were: illiterate conversational speech rate and delta-theta PSI, r = -.326, n = 15, p = .236; low literate conversational speech rate and delta-theta PSI, r = -.308, n = 19, p = .200; high literate conversational speech rate and delta-theta PSI, r = -.023, n = 12, p = .944. The theta-beta/low gamma PSI correlations were illiterate conversational speech rate and theta-beta/low gamma PSI, r = -.428, n = 15, p = .112; low literate conversational speech rate and theta-beta/low gamma PSI, r = .086, n = 19, p = .725; high literate conversational speech rate and theta-beta/low gamma PSI, r = -.126, n = 12, p = .696.

We had predicted that any temporal modulation differences between literate and illiterate speech should relate also to phoneme awareness. As noted earlier, we had measured speech comprehension (Token Test), vocabulary (WAIS) and phonological awareness of syllables, rhymes and phonemes in our participants (see Table 1), enabling individual differences to be correlated with the modulation spectrum and phase synchronisation measures. As expected [6] the illiterate participants could not perform the phoneme awareness task. Since the distribution of language task scores did not reveal an approximately normal distribution (p’s < 0.05 using Shapiro-Wilk tests), non-parametric tests were conducted to compare the differences in performances between groups. Kruskal-Wallis tests showed a significant effect of group for each task (all p’s < 0.0001). Subsequent exploration via Mann-Whitney tests showed that high literates performed better than low literates and that low literates performed better than illiterates in all tasks (all p’s < 0.013).

Correlations with the speech production measures are shown in Table 2. We ran Spearman’s correlations by rank; scatterplots relating to the correlations for rhyme awareness and vocabulary with the PSI measures are supplied for illustrative purposes as Supplementary Materials (S1–S4 Figs). Contrary to prediction, the Table shows that the amount of delta-band energy was not correlated with literacy, however as predicted the amount of theta-band energy was significantly related to literacy. Thus the more years of literacy tuition, the more theta-band energy in spontaneous conversational speech. More theta-band energy was also found in the speech of participants with better vocabulary knowledge. As speech rate was also positively related to theta-band energy, it is likely that these 3 factors are inter-dependent: the more literate participants have larger vocabularies and consequently are likely to produce more complex syntax and more polysyllabic words, resulting in more syllables per second. The uniformly positive correlations between phase synchronisation and the phonology and language tasks suggest that stronger phase synchronisation between different phonological units in speech is related to better metaphonological skills, which would be expected. After applying Bonferroni corrections for multiple comparisons, most of these relations remained significant. Participants with more years of literacy showed significantly greater delta-theta phase alignment, and greater delta-theta phase alignment was related to better syllable and phoneme skills, and better speech comprehension (as measured by the Token Test). Greater theta-beta/low gamma phase alignment was significantly related to better rhyme detection and better vocabulary. As would be expected from our prior analyses, speech rate did not show a relationship to phase synchronisation, and nor did age. As the relationships found for theta band modulation energy did not survive Bonferroni correction, they should be considered suggestive only.

**Table 2:** Correlations between modulation energy and AM phase synchronisation and the behavioural tests for conversational speech.

	Delta AM Energy	Theta AM Energy	Delta-Theta PSI	Theta-Beta/Low gamma PSI
**Literacy (years)**	-0.16	0.30*	0.46***	0.43**
**Syllabic division**	-0.05	0.17	0.46***	0.38**
**Rhyme detection**	-0.00	0.13	0.41**	0.45***
**Phoneme deletion**	-0.15	0.26	0.51***	0.39**
**Vocabulary (WAIS-III)**	-0.20	0.33*	0.35**	0.45***
**Token Test (*n = 45*)**	-0.05	0.22	0.48***	0.43**
Control variables				
**Speech Rate**	-0.28	0.29*	-0.11	0.05
**Age**	-0.05	0.11	-0.21	-0.24

*** p ≤ .0021

** p ≤ .01

* p≤ .05.

## Discussion

Our study is the first to show that the temporal modulation structure of the speech of adults is not equal. Adults who have never learned to read produce everyday conversational speech that has significantly less phase alignment (less synchronisation) between the slower AM bands, delta and theta, than the speech of adults with either low or high levels of literacy. These group effects for delta-theta phase alignment were found across the spectral range of speech (100 Hz– 7250 Hz). There was a phase synchronisation effect for faster AM bands also, but here the effect was significant in the lowest frequency band only (100–300 Hz). The same effect of literacy was found, with greater theta-beta/low gamma band phase synchronisation for the two literate groups compared to the illiterate group. The difference between illiterates and low literates shows that even a few years of literacy tuition (≤ 4 years) has an effect on spoken language. Greater phase synchronisation is suggestive of a more tightly controlled spacing of different phonological units in words. Greater theta-beta/low gamma band phase synchronisation is suggestive of more regular spacing of phonemes within syllables, while greater delta-theta phase synchronisation is suggestive of more regular spacing regarding syllables and stressed syllables. The degree of phase alignment between both slower and faster AM bands in conversational speech was also significantly related to speech comprehension (Token Test), vocabulary development and phonological awareness (syllable, rhyme and phoneme measures). While the effects of literacy on speech perception are relatively well-documented [6,7,9–16], to our knowledge our study is the first to demonstrate an effect of literacy on conversational speech. Hence cultural skills affect speech production.

Years of literacy showed a positive relationship with the amount of theta-band modulation energy in conversational speech, although this correlation did not survive Bonferroni correction. The positive relationship between years of literacy and theta band modulation energy is consistent with data from typically-developing children, for whom the strength of theta entrainment (neural measure) is significantly correlated with literacy [25]. These data suggest that the key role identified for theta-rate information in adult speech processing may indeed be, at least in part, dependent on literacy development. The data suggest that increased phase alignment between slower rhythmic information in speech (delta- and theta-band AMs) and possibly increased theta-band modulation energy are markers of literacy-dependent acoustic changes in the way that speech is produced.

Notably, the rhythmic speech of illiterate adults was not different to the rhythmic speech of low or high literate adults. These data are important. They suggest that there is no physiological constraint governing the differences in conversational speech found for the illiterate group. The rhythmic speech data also present an interesting contrast to the case of dyslexic adults [22]. In [22], dyslexic adults could not produce deliberately rhythmic speech with the same temporal characteristics as non-dyslexic literate adults. In contrast, illiterate adults could choose to deliberately produce rhythmic speech, and their rhythmic speech production had the same temporal characteristics as the rhythmic speech of literate adults.

Although the significant relationships found between the degree of delta-theta phase synchronisation and syllable and rhyme awareness may be expected, the significant correlation with *phoneme awareness* may appear surprising. In multi-time resolution models of speech processing, faster oscillatory information is thought to support phoneme awareness (specifically, gamma band information, see [3]). However, recent modelling studies suggest that both rhyme awareness and phoneme awareness are related specifically to delta- and theta-band AM information. A recent study of the phoneme deletion metaphonological task from an AM perspective revealed that the consistent acoustic correlate of phoneme deletion was the magnitude of the change in delta-theta AM phase synchronisation from target to response [42]. Changes in the magnitude of theta-beta/low gamma AM band phase synchronisation did not relate in any systematic way to phoneme deletion in this study. Similarly, a recent AM-motivated study of rhyme awareness found that rhyme similarity was governed by delta-band AM phase information [43]. Accordingly, the role of slower AMs in neural speech encoding, speech production and phonological awareness are deserving of further study.

One limitation of the current study is the relatively old age of the participants, who were screened for general cognitive impairments (via the MMSE) but not specifically matched for hearing. It could be important to access other illiterate populations, ideally younger than the population studied here. This would also enable investigation of whether the growing literature on the core role of theta entrainment for neural speech encoding [3,5] is an epiphenomenon of the continued experimental study of highly literate participants.

In conclusion, our data suggest that future neural imaging studies of features of spoken language performance in both perception and production must take into account the literacy skills of participants. Literacy has long been known to change the brain [12,13,16]. Here we show that it also affects the temporal characteristics of spoken language.

## Supporting information

S1 Fig Scatterplot of rhyme awareness and delta-theta PSI correlation. (TIF)

S2 Fig Scatterplot of rhyme awareness and theta-beta/low gamma PSI correlation. (TIF)

S3 Fig Scatterplot of vocabulary and delta-theta PSI correlation. (TIF)

S4 Fig Scatterplot of vocabulary and theta-beta/low gamma PSI correlation. (TIF)
