# Supplementary figures and images for: The temporal modulation structure of illiterate versus literate adult speech
Source: PLoS One. 2018 Oct 24;13(10):e0205224. doi: 10.1371/journal.pone.0205224 (PMC6200213; doi:10.1371/journal.pone.0205224)

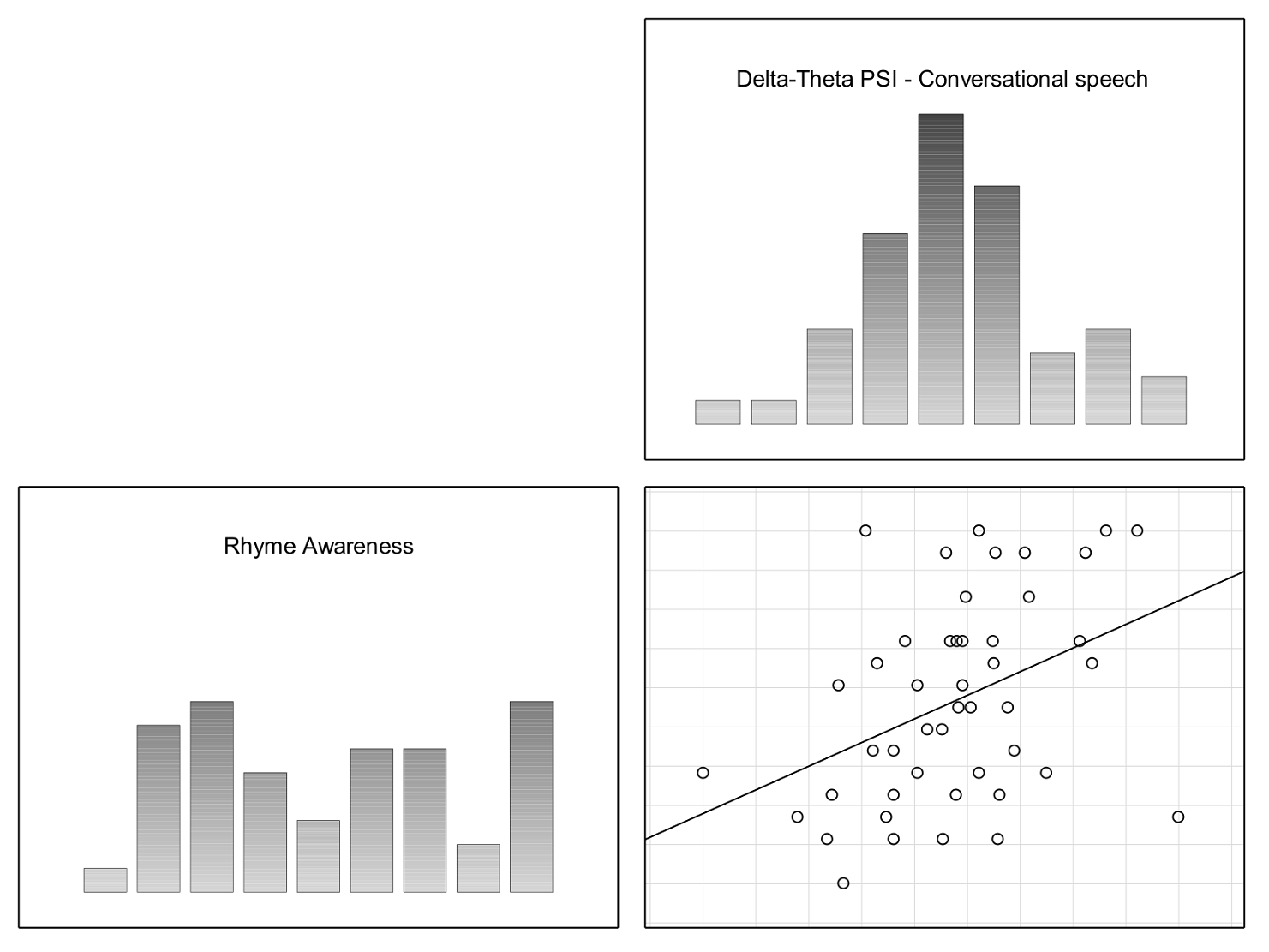

Supplement: S1 Fig — (TIF) [file pone.0205224.s001.tif]

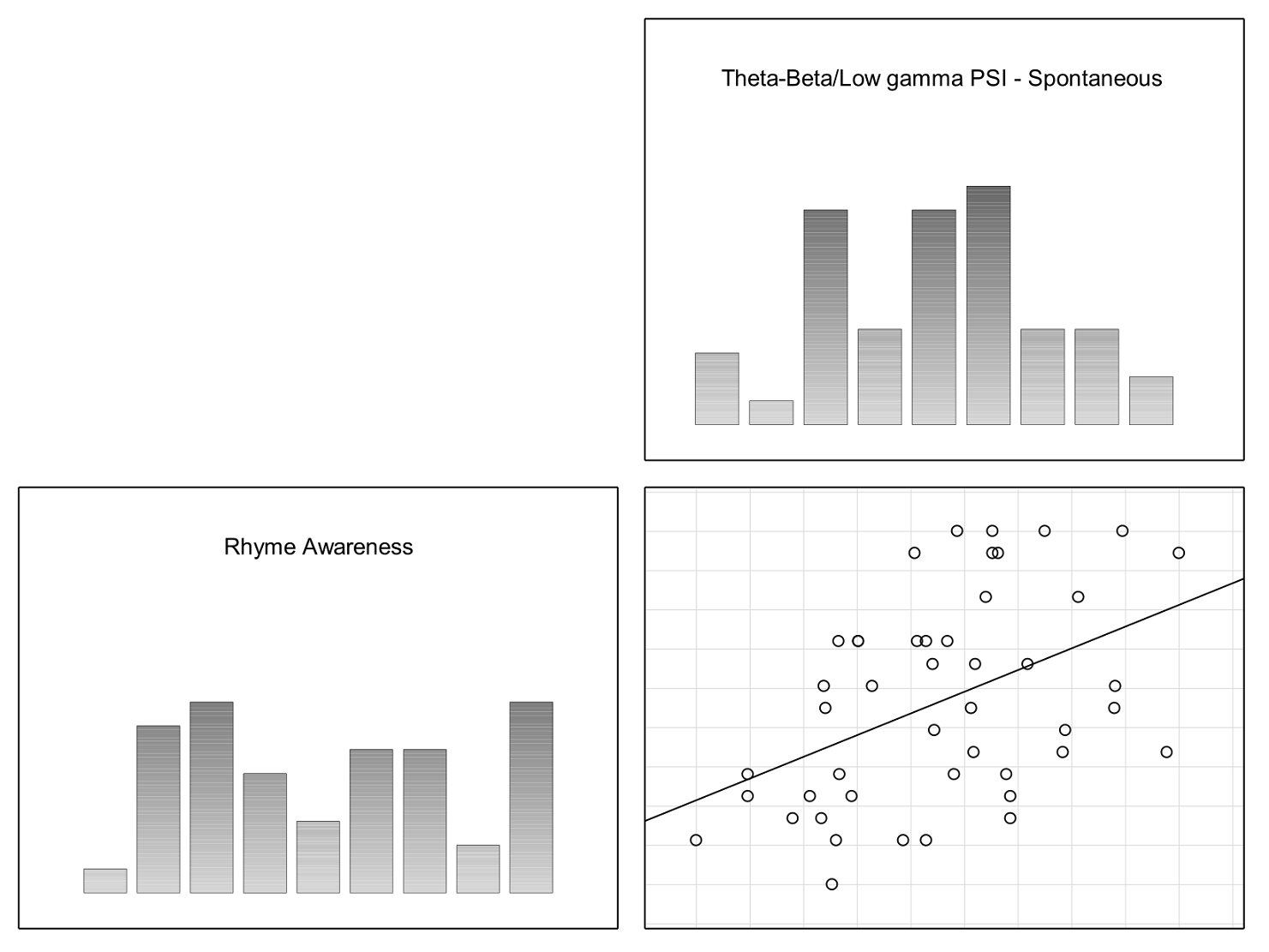

Supplement: S2 Fig — (TIF) [file pone.0205224.s002.tif]

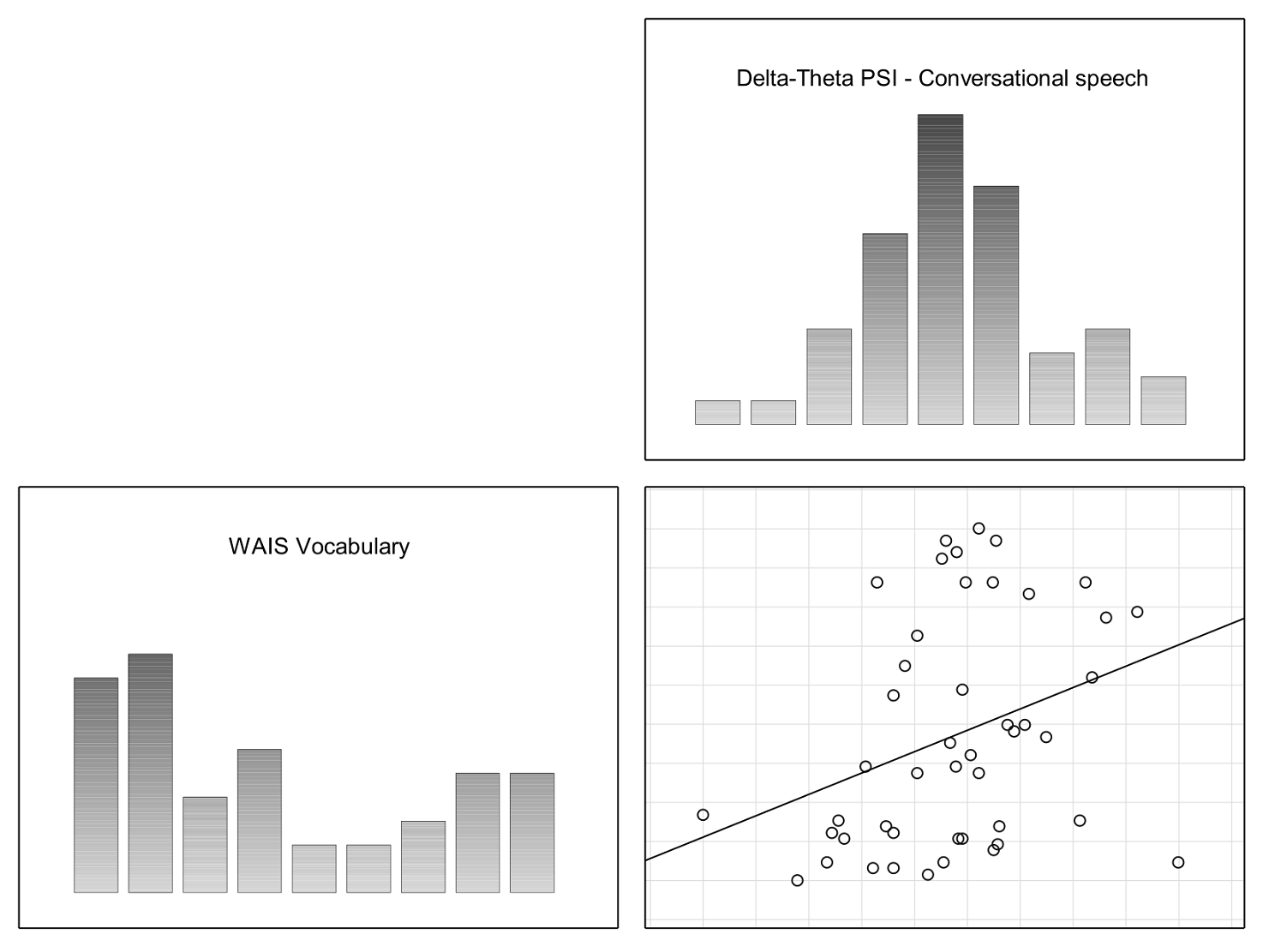

Supplement: S3 Fig — (TIF) [file pone.0205224.s003.tif]

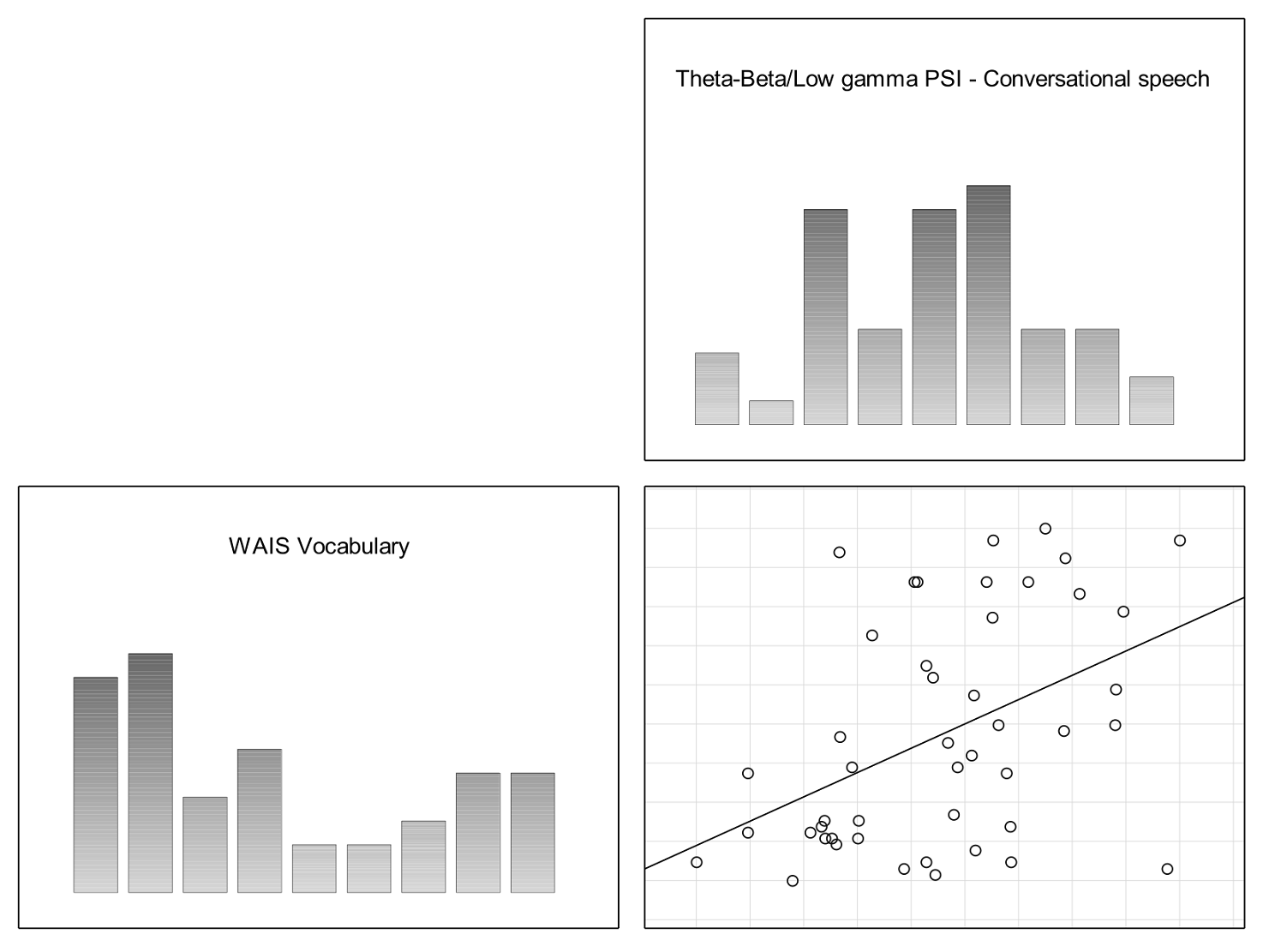

Supplement: S4 Fig — (TIF) [file pone.0205224.s004.tif]
